# Fear of COVID-19 and Trust in the Healthcare System Mediates the Association between Individual’s Risk Perception and Preventive COVID-19 Behaviours among Iranians

**DOI:** 10.3390/ijerph182212146

**Published:** 2021-11-19

**Authors:** Mehran Alijanzadeh, Daniel Kwasi Ahorsu, Zainab Alimoradi, Narges Mahmoudi, Mark D. Griffiths, Chung-Ying Lin, Hsien-Kuan Liu, Amir H. Pakpour

**Affiliations:** 1 Social Determinants of Health Research Center, Research Institute for Prevention of Non-Communicable Diseases, Qazvin University of Medical Sciences, Qazvin 3419759811, Iran; mehran_alijanzade@yahoo.com (M.A.); zainabalimoradi@yahoo.com (Z.A.); mahmoudi.narges@yahoo.com (N.M.); 2 Department of Rehabilitation Sciences, Faculty of Health & Social Sciences, The Hong Kong Polytechnic University, 11 Yuk Choi Rd Hung Hom, Hong Kong, China; daniel.ahorsu@connect.polyu.hk; 3 Psychology Department, Nottingham Trent University, Nottingham NG3 5DS, UK; mark.griffiths@ntu.ac.uk; 4 Institute of Allied Health Sciences, College of Medicine, National Cheng Kung University, Tainan 701401, Taiwan; cylin36933@gmail.com; 5 Department of Pediatrics, E-DA Hospital, Kaohsiung 82445, Taiwan; 6 School of Medicine, I-Shou University, Kaohsiung 840203, Taiwan; 7 Department of Nursing, School of Health and Welfare, Jönköping University, 55111 Jönköping, Sweden

**Keywords:** COVID-19, fear of COVID-19, trust in the healthcare system, risk perception, preventive COVID-19 behaviours

## Abstract

Problems caused by the novel coronavirus disease-2019 (COVID-19) and its mutations have brought challenges in pandemic control for all countries worldwide. The present study examines the mediating roles of fear of COVID-19 and trust in the healthcare system in the association between individual’s risk perception and performing preventive COVID-19 behaviours among Iranians. A cross-sectional study design was used to collect data from 3652 residents of Qazvin province in Iran from 3 February to 15 April 2021 using a multistage stratified cluster sampling method. Participants responded to an online questionnaire concerning their fear of COVID-19, risk perception, trust in the healthcare system, and preventive COVID-19 behaviours. Small to medium positive interrelationships were observed between the variables of the study. Fear of COVID-19, trust in the healthcare system or both (fear of COVID-19 and trust in the healthcare system) mediated the association between an individual’s risk perception and performing preventive COVID-19 behaviours. The study demonstrated there are at least four ways through which an individual’s risk perception can influence preventive COVID-19 behaviours. Therefore, clinicians, health communicators, and researchers may capitalize on these findings to enhance preventive COVID-19 behaviours to help mitigate the spread of COVID-19 infection.

## 1. Introduction

The novel coronavirus disease 2019 (COVID-19) has spread rapidly around the world and has infected many individuals in countries around the world [1,2]. Globally (at the time of writing), there have been over 253 million COVID-19 cases and over 5.1 million deaths, while over 6.03 million cases and over 128,000 deaths have been reported among Iranians (the location of the present study) [3]. The World Health Organisation has repeatedly emphasised a range of interventions to control the spread of COVID-19 and reduce the risk of transmission by avoiding direct contact with infected people, washing hands frequently, wearing masks, and physical distancing [4]. In closed environments, emphasis on proper ventilation and air exchange is essential to preventing COVID-19 [5]. However, since studies on treatment and preventive methods have not yet reached definitive conclusions, adherence to preventive measures and published guidelines is the only sensible strategy to control the disease [6,7].

Considering that the prevalence of this disease is not specific to any age group (although older aged individuals are more vulnerable to experiencing serious consequences), informing the general public and different groups through short-term health education programmes and providing health messages can be effective in reducing the COVID-19 burden [8,9]. Research findings have shown that individuals’ adherence to preventive measures is greatly influenced by their knowledge and awareness of risk factors associated with infectious and contagious diseases [10]. The results of previous research have shown that psychological factors, such as perceived susceptibility to and perceived severity of diseases, can be useful in controlling and reducing the impact of an epidemic as well as engaging in protective and preventive behaviours among individuals [11]. Currently, there is evidence that individuals’ fear of COVID-19 and their perceptions of vulnerability to the disease lead to preventive behaviours [12,13].

Therefore, individuals’ fear of COVID-19 and being infected by this disease leads individuals to perform more preventive behaviours and may make them follow instructions provided to deal with the problem [14]. Yıldırım et al. [15] recently showed that there appears to be a direct link between fear of COVID-19 and performing more preventive measures, such that those individuals who feel fear adhere more to preventive behaviours. A study conducted by Breakwell et al. [16] in the UK also showed that in addition to preventive measures such as wearing a mask and washing hands, individuals should prioritize a distance of two metres from each other, eliminate unnecessary travel, eliminate unnecessary visits, and follow the latest COVID-19 guidelines [16].

Chan et al. [17] examined the key issue of public trust in the healthcare system. The results of their study suggested that there was a relationship between trust in the healthcare system and performing preventive COVID-19 behaviours. Their study showed that individuals with higher trust in the healthcare system were better at performing preventive COVID-19 behaviours. Therefore, trust in the health system plays an essential role in all medical relationships and an important role in positive treatment outcomes as a predictor [18,19]. Individuals’ distrust of the healthcare system and consequent decrease in physician-patient interactions leads to weaker clinical relationships. This reduces adherence to recommendations and reduces referral to the healthcare system. Therefore, higher trust among service providers in healthcare systems will lead to performing more preventive COVID-19 behaviours among individuals [20,21,22]. Consequently, the present study examined the (i) relationship between four key variables (risk perception, fear of COVID-19, trust in the healthcare system, and preventive COVID-19 behaviours), and the (ii) mediating roles of fear of COVID-19 and trust in the healthcare system in the association between an individual’s risk perception and performing preventive COVID-19 behaviours.

## 2. Materials and Methods

### 2.1. Participants and Procedure

A cross-sectional survey study was conducted from 3 February to 15 April 2021 among residents in Qazvin province, Iran. Study participants were recruited from the Integrated Health System (IHS: SIB in Persian). This system is a comprehensive system for the electronic registration of all households in Iran as well as the registration of all health services that individuals receive from various medical departments in health centres. This system has full access to the home address and home phone number of all Iranian individuals. The study’s aim was explained to the participants, and they were assured that their information would remain confidential. Participants were free to participate in the present study and if they did not want to, they could withdraw from the study at any time. A total of 4500 individuals were randomly selected from the IHS. A Short Messaging Service (SMS) text containing the link to the study’s measures and information was sent to them once only. Those who provided their online consent were invited to complete the survey, which took approximately 20 minutes to complete. The inclusion criteria were all residents who lived in Qazvin province and being an adult (18 years and older). The exclusion criteria were residents who were minors (i.e., 17 years and below) and those who were not Iranian. All procedures were carried out in compliance with the Helsinki Declaration. Additionally, participants were assured of the confidentiality and anonymity of their responses, as well as their right to withdraw from the study at any time without any consequences. No remuneration or rewards were given for participation in the study, although participants were sincerely thanked for taking part at the end of the survey. The Qazvin University of Medical Sciences Ethics Committee approved the study protocol (reference number IR.QUMS.REC.1399.458, approved 6 February 2021).

### 2.2. Measures

#### 2.2.1. Preventive Behaviour Scale (PBS)

The PBS was developed based on the five WHO-recommended preventive behaviours, and items are rated on a seven-point scale ranging from 1 (not at all) to 7 (always) to determine a person’s degree of performing preventive behaviour. Higher scores indicate greater adherence to the WHO recommendations in relation to preventing the spread of COVID-19 [23]. Sample items include “Regularly and thoroughly clean your hands with an alcohol-based hand rub or wash them with soap and water”. Before responding to the scale, participants were given a clear definition of preventive COVID-19 behaviours and recommendations of how and when to perform them according to WHO guidelines [24]. Therefore, participants were fully aware of the definitions of preventive behaviours and guidelines. The Cronbach’s alpha coefficient for the present study was 0.792 (see Supplementary Table S1 for details). This scale has been used previously among Iranians [25,26].

#### 2.2.2. Fear of COVID-19 Scale (FCV-19S)

To assess respondents’ fear of COVID-19, the FCV-19S was used. The FCV-19S is a self-administered measure with seven items that are rated on a five-point scale ranging from 1 (strongly disagree) to 5 (strongly agree). Sample items include “It makes me uncomfortable to think about coronavirus-19”. The total score is obtained by summing up all responses to the items, with higher scores indicating greater fear [27,28]. The Cronbach’s alpha coefficient for the present study was 0.886 (see Supplementary Table S1 for details). This scale has previously been used among Iranians [25,28].

#### 2.2.3. Risk Perception Scale (RPS)

The three-item RPS was used to assess participants’ perception of the risk of getting COVID-19. All items are rated on a five-point scale ranging from 1 (strongly disagree) to 5 (strongly agree). Sample items include “In general, how serious do you think COVID-19 is?” [15]. The Cronbach’s alpha coefficient for the present study was 0.768 (see Supplementary Table S1 for details).

#### 2.2.4. Revised Health Care System Distrust Scale (RHCSDS)

Participants’ trust in the healthcare system was assessed using the RHCSDS. The nine-item RHCSDS assesses individuals’ perception of honesty, confidentiality, competence, and fidelity in the healthcare system. All items are rated on a five-point scale ranging from 1 (strongly disagree) to 5 (strongly agree), with higher scores indicating greater trust in the healthcare system. Sample items include “The healthcare system covers up its mistakes” [29]. The Cronbach’s alpha coefficient for the present study was 0.818 (see Supplementary Table S1 for details).

#### 2.2.5. Sociodemographic Characteristics

The last section of the survey included questions related to age, gender, educational status, marital status, and living accommodation.

### 2.3. Data Analysis

The relationships between all variables of interest (risk perception, fear of COVID-19, trust in the healthcare system, and preventive COVID-19 behaviours) were examined using Pearson correlation coefficients. For the serial multiple mediation model, risk perception was the independent variable, fear of COVID-19 and trust in the healthcare system were the mediators, and preventive COVID-19 behaviours was the dependent variable. Age, gender, educational status, marital status and living accommodation were all adjusted for in the mediation model, as they have all been found in previous research to have an influence on the results [30]. The PROCESS macro for SPSS (version 25) (IBM, Armonk, NY, USA) was used for the mediation analysis using Model 6 and 10,000 bootstrapping resamples (Model 6, Process Macro) [31]. Moreover, all the instruments used in the present study had their construct validity evaluated using confirmatory factor analysis using the diagonally weighted least squares estimator in R software with the *lavaan* package [32]. The reliability of all the measures used in the present study was examined using Cronbach’s α, McDonald’s ω coefficients, and the composite reliability (CR) coefficient. Coefficients exceeding 0.70 for α and ω and 0.60 for CR were considered adequate for the internal consistency of the respective statistic. Additionally, a coefficient exceeding 0.50 was considered satisfactory for the average variance extracted (AVE) so as to confirm that items of each latent variable contributed adequately to that construct [26].

## 3. Results

A total of 3652 participants completed the survey in the present study, with a mean age of 34.15 years (SD = 12.31). The majority of the participants were female (*n* = 2558, 70%), university students (*n* = 1807, 50%), married (*n* = 2759, 76%), and living in urban areas (*n* = 2931, 80%) (see Table 1 for more details).

Preliminary analysis showed that factor loadings, reliability coefficients, and AVE were all within the acceptable limits. More specifically, Cronbach’s α and McDonald’s ω coefficients were all above 0.70, CR was above 0.60, and AVE values were about 0.50, all of which support the internal consistency and reliability of the measures used in the present study (see Supplementary Table S1). Table 2 shows the descriptive statistics of the various measures used in the present study according to the demographic characteristics of the participants. It was found that females had slightly higher scores than males on risk perception, fear of COVID-19, trust in the healthcare system, and preventive COVID-19 behaviours. There were between-group differences in the various educational levels on all the measures used. This also applied to marital status and whether participants had children or not. However, it must be noted that these differences were just descriptive. Therefore, they did not necessarily indicate statistically significant differences.

The correlation analysis showed that there were positive associations between fear of COVID-19 and risk perception (*r* = 0.321, *p* < 0.01), fear of COVID-19 and trust in the healthcare system (*r* = 0.170, *p* < 0.01), fear of COVID-19 and preventive COVID-19 behaviours (*r* = 0.125, *p* < 0.01), risk perception and trust in the healthcare system (*r* = 0.110, *p* < 0.01), risk perception and preventive COVID-19 behaviours (*r* = 0.091, *p* < 0.01), and trust in the healthcare system and preventive COVID-19 behaviours (*r* = 0.228, *p* < 0.01) (see Table 3 for details).

**Figure 1:**
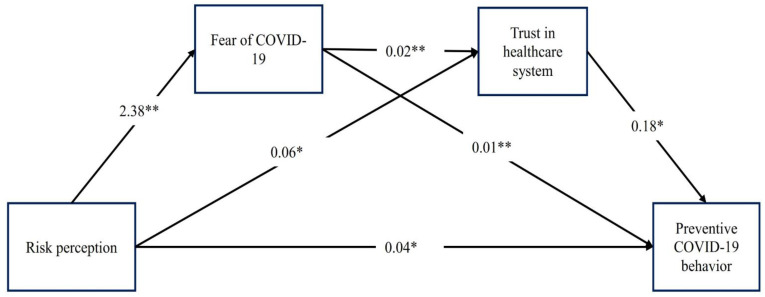
The serial multiple mediation model with fear of COVID-19 and trust in the healthcare system as proposed mediators of the effect of risk perception on performing preventive COVID-19 behaviours. * *p* < 0.05. ** *p* < 0.001. Note: Age, gender, educational status, marital status and living accommodation were adjusted in the model.

The mediation analysis showed that fear of COVID-19 (unstandardized coefficient = 0.020; LLCI = 0.010; ULCI = 0.030), trust in the healthcare system (unstandardized coefficient = 0.011; LLCI = 0.004; ULCI = 0.018), and both fear of COVID-19 and trust in the healthcare system (unstandardized coefficient = 0.008; LLCI = 0.006; ULCI = 0.0012) were significant mediators in the association between risk perception and preventive COVID-19 behaviours. Therefore, the total indirect effect (0.039) was significant (LLCI = 0.027; ULCI = 0.052). Additionally, there were significant direct effects of risk of perception on preventive COVID-19 behaviours (unstandardized coefficient of 0.036; SE = 0.014; *p* = 0.011) with a significant total effect on preventive COVID-19 behaviours (unstandardized coefficient of 0.075; SE = 0.014; *p* < 0.001) (see Table 4 and Figure 1 for details).

## 4. Discussion

The present cross-sectional study examined the (i) relationships between an individual’s risk perception, fear of COVID-19, trust in the healthcare system and preventive COVID-19 behaviours, and (ii) the mediating roles of the fear of COVID-19 and trust in the healthcare system in the association between an individual’s risk perception and performing COVID-19 preventive behaviours. The correlation results showed that there were significantly positive associations between all the variables, with small to medium effects [33,34], which indicated that when one variable increased, the other also increased and vice versa. These findings are in line with previous findings reporting an association between fear of COVID-19, risk perception, and preventive COVID-19 behaviours [14,15,25,26].

The serial multiple mediation results showed that there was a significant direct association between an individual’s risk perception and preventive COVID-19 behaviours. This suggests that risk perception directly relates to preventive COVID-19 behaviours, further implying that higher risk perception may influence higher preventive COVID-19 behaviours and vice versa. In addition, there were significant indirect associations between an individual’s risk perception and preventive COVID-19 behaviours via either fear of COVID-19, trust in the healthcare system or both (fear of COVID-19 and trust in the healthcare system). That is, apart from risk perception directly relating to preventive COVID-19 behaviours, it may also relate through three different paths: (i) fear of COVID-19, (ii) trust in the healthcare system, or (iii) both (fear of COVID-19 and trust in the healthcare system). More specifically, there were at least four ways in which risk perception as the sole agent or in combination with (i) fear of COVID-19, (ii) trust in the healthcare system, or (iii) both (fear of COVID-19 and trust in the healthcare system) may influence preventive COVID-19 behaviours. This implies that there are at least four ways in which health authorities or researchers can help to control an individual’s risk perception to influence their preventive COVID-19 behaviours based on the findings of this study. However, this does not exclude the possible roles of other variables that may influence COVID-19 preventive behaviours (e.g., family opinions, cultural and religious beliefs). These findings are similar to previous studies that have used different participants or conditions [10,11,12,13].

The findings in the present study are comforting, because they suggest that there are multiple ways through which risk perception can influence preventive COVID-19 behaviours. Therefore, clinicians, health communicators, and researchers may utilize these findings to enhance preventive COVID-19 behaviours to help mitigate the spread of COVID-19 infection. However, future studies should also investigate other variables that may help with preventive COVID-19 behaviours in order to develop multifactorial models to help fight COVID-19.

### Limitations

The present study has some limitations. First, the present study used a cross-sectional design which, at best, provided only strong associations between the variables and not cause and effect relationships. Therefore, the findings should be applied with caution and additional longitudinal and/or randomized control trial studies need be conducted to further examine these variables. Second, the participants of the present study can be categorized as younger aged adults. Therefore, the findings cannot be generalized to all adults. Future studies may consciously recruit older adults to ascertain a more holistic picture of the variables studied. Third, the sample was predominantly urban, female, well educated, and married, and so cannot be generalized to the Iranian population as a whole or to other countries and cultures worldwide. Therefore, future research needs to overcome these limitations and include samples that are as representative as possible within their own country. Fourth, the present study gathered data using self-report measures, which may be susceptible to biases including social desirability. However, the self-report instruments have robust psychometric properties. This suggests that the data are valid and trustworthy to some degree.

## 5. Conclusions

The present study demonstrated positive associations between risk perception, fear of COVID-19, trust in the healthcare system, and preventive COVID-19 behaviours as well as the mediating effects of fear of COVID-19 and trust in the healthcare system in the association between risk perception and performing COVID-19 preventive behaviours. It was also observed that risk perception, directly and indirectly, influenced preventive COVID-19 behaviours. Additionally, perception of the risk of getting COVID-19 significantly and positively influenced individuals’ fear of COVID-19 and trust in healthcare systems, which helps influence preventive COVID-19 behaviours. These findings suggest that there are multiple ways in which risk perception can influence preventive COVID-19 behaviours. Therefore, appropriate presentation of the risk of contracting COVID-19 by healthcare experts and communicators, which increases fear of COVID-19, may go a long way to improving preventive COVID-19 behaviours. Therefore, clinicians, health communicators, and researchers may utilize the findings here to enhance preventive COVID-19 behaviours to help mitigate the spread of COVID-19 infection.

## Figures and Tables

**Table 1:** Participants’ sociodemographic characteristics (n = 3652).

	Mean (SD) or *n* (%)
Age (year)	34.15 (±12.31)
Gender	
*Male*	1094 (30%)
*Female*	2558 (70%)
Educational status	
*Primary school*	195 (5%)
*Secondary school*	759 (21%)
*Diploma*	891 (24%)
*University*	1807 (50%)
Marital status	
*Single*	893 (24%)
*Married*	2759 (76%)
Accommodations	
*Rural*	721 (20%)
*Urban*	2931 (80%)

**Table 2:** Means and standard deviations of the measures according to the sociodemographic characteristics.

		Risk Perception	Fear of COVID-19	Trust in the Healthcare System	Preventive COVID-19 Behaviours
Gender	Male	4.44 ± 0.77	19.29 ± 6.89	3.88 ± 0.85	6.02 ± 0.92
	Female	4.60 ± 063	21.68 ± 6.85	3.94 ± 0.79	6.38 ± 0.68
Educational status	Primary school	3.11 ± 0.95	20.54 ± 8.05	3.69 ± 0.99	6.31 ± 0.95
	Secondary school	2.96 ± 0.95	20.97 ± 7.20	3.87 ± 0.84	6.15 ± 0.87
	Diploma	3.15 ± 0.97	21.55 ± 6.97	3.91 ± 0.91	6.34 ± 0.70
	University	3.22 ± 0.90	20.73 ± 6.70	3.85 ± 0.89	6.29 ± 0.75
Marital status	Single	4.42 ± 0.79	19.40 ± 7.12	3.84 ± 0.85	4.42 ± 0.79
	Married	3.95 ± 0.79	21.47 ± 6.82	4.59 ± 0.64	6.32 ± 0.72
Accommodation	Rural	4.46 ± 0.69	20.14 ± 7.25	3.99 ± 0.74	6.11 ± 0.90
	Urban	4.56 ± 0.68	21.17 ± 6.86	3.91 ± 0.82	6.31 ± 0.73

**Table 3:** Correlation matrix of the study’s variables.

	Fear of COVID-19	Risk Perception	Trust in Healthcare System	Preventive COVID-19 Behaviours	Mean	SD	Range
Fear of COVID-19	-	0.321 **	0.170 **	0.125 **	21.00	6.95	7–35
Risk perception	-	-	0.110 **	0.091 **	3.14	0.94	1–5
Trust in healthcare system	-	-	-	0.228 **	3.88	0.89	1–5
Preventive COVID-19 behaviours	-	-	-	-	6.27	0.78	1–7

** *p* < 0.01.

**Table 4:** Models of the effect of risk perception on adherence to preventive COVID-19 behaviours with mediators of fear of COVID-19 and trust in healthcare system.

Model	Coefficient	SE	*t*	*p*
Total effect of risk perception on preventive COVID-19 behaviours	0.075	0.014	5.493	<0.001
Direct effect of risk perception on preventive COVID-19 behaviours in mediated model	0.036	0.014	2.559	0.0105
Indirect effect of risk perception on preventive COVID-19 behaviours	Effect	Boot SE	Boot LLCI	Boot ULCI
Total indirect effect	0.039	0.007	0.027	0.052
Indirect effect via fear of COVID-19	0.020	0.005	0.010	0.030
Indirect effect via trust in healthcare system	0.011	0.003	0.004	0.018
Indirect effect via fear of COVID-19 and trust in healthcare system	0.008	0.001	0.006	0.012

## Data Availability

Data sharing not applicable.

## Supplementary Materials

The following are available online at https://www.mdpi.com/article/10.3390/ijerph182212146/s1. Table S1: Items for all study measures with descriptive statistics, reliability coefficients, factor loadings, and average variance extracted statistics.
