# Supplementary material for: Fear of COVID-19 and Trust in the Healthcare System Mediates the Association between Individual’s Risk Perception and Preventive COVID-19 Behaviours among Iranians
Source: Int J Environ Res Public Health. 2021 Nov 19;18(22):12146. doi: 10.3390/ijerph182212146 (PMC8621944; doi:10.3390/ijerph182212146)
Supplement: Supplementary file 1 [file ijerph-18-12146-s001.zip › ijerph-1436392-supplementary.pdf]

Supplementary Analyses

Table S1. Items for all study measures with descriptive statistics, reliability coefficients, factor loadings, and average variance extracted statistics

Construct; Mean (SD)	Measurement item	λ	α	ω	CR	AVE
Trust	The healthcare system makes patients' health better	0.750	0.818	0.803	0.88	0.46
	The healthcare system covers up its mistakes	0.522				
	Patients receive high-quality care from the healthcare system	0.648				
	The healthcare system makes too many mistakes	0.793				
	The healthcare system puts making money above patients' needs	0.689				
	The healthcare system gives excellent medical care.	0.728				
	Patients get same medical treatment regardless of race	0.552				
	The healthcare system lies to make money	0.774				
	The healthcare system experiments on patients without them knowing	0.580				
Perceived risk	Compared to most people your age and sex, what would you say your chances are for developing coronavirus?	0.670	0.768	0.776	0.746	0.495
	How risky would it be for you if you think that you have coronavirus symptoms?	0.709				
	In general, how serious do you think COVID-19 is?	0.731				
Preventive COVID-19 behaviors	Regularly and thoroughly clean your hands with an alcohol-based hand rub or wash them with soap and water	0.712	0.792	0.816	0.877	0.591
	Practice respiratory hygiene (covering your mouth and nose with your bent elbow or tissue when you cough or sneeze)	0.730				
	Maintain at least one meter (3 feet) distance between yourself and anyone	0.640				

	who is coughing or sneezing					
	Stay home if you feel unwell	0.866				
	Avoid touching eyes, nose, and mouth	0.872				
Fear	I am most afraid of coronavirus-19	0.657	0.886	0.884	0.881	0.516
	It makes me uncomfortable to think about coronavirus-19.	0.703				
	My hands become clammy when I think about coronavirus-19	0.710				
	I am afraid of losing my life because of coronavirus-19	0.682				
	When watching news and stories about coronavirus-19 on social media, I become nervous or anxious	0.743				
	I cannot sleep because I'm worrying about getting coronavirus-19	0.751				
	My heart races or palpitates when I think about getting coronavirus-19	0.778				

Note. λ = Standardized factor loading from structural equation model; α = Cronbach's alpha reliability coefficient; ω = McDonald's omega reliability coefficient; CR = Composite reliability; AVE = Average variance extracted from structural equation model.
